# Ibuprofen arginate retains eNOS substrate activity and reverses endothelial dysfunction: implications for the COX-2/ADMA axis

**DOI:** 10.1096/fj.201600647R

**Published:** 2016-09-06

**Authors:** Nicholas S. Kirkby, Abel Tesfai, Blerina Ahmetaj-Shala, Hime H. Gashaw, Walkyria Sampaio, Gisele Etelvino, Nádia Miricéia Leão, Robson A. Santos, Jane A. Mitchell

**Affiliations:** * Vascular Biology Section, National Heart and Lung Institute, Imperial College London, London, United Kingdom; and; † Department of Physiology and Biophysics, National Institute in Science and Technology in Nanobiopharmaceutics, Federal University of Minas Gerais, Belo Horizonte, Brazil

**Keywords:** aspirin, methylarginines, nitric oxide, prostacyclin, Vioxx

## Abstract

Nonsteroidal antiinflammatory drugs, including ibuprofen, are among the most commonly used medications and produce their antiinflammatory effects by blocking cyclooxygenase (COX)-2. Their use is associated with increased risk of heart attacks caused by blocking COX-2 in the vasculature and/or kidney, with our recent work implicating the endogenous NOS inhibitor asymmetric dimethylarginine (ADMA), a cardiotoxic hormone whose effects can be prevented by l-arginine. The ibuprofen salt ibuprofen arginate (Spididol) was created to increase solubility but we suggest that it could also augment the NO pathway through codelivery of arginine. Here we investigated the idea that ibuprofen arginate can act to simultaneously inhibit COX-2 and preserve the NO pathway. Ibuprofen arginate functioned similarly to ibuprofen sodium for inhibition of mouse/human COX-2, but only ibuprofen arginate served as a substrate for NOS. Ibuprofen arginate but not ibuprofen sodium also reversed the inhibitory effects of ADMA and *N*^G^-nitro-l-arginine methyl ester on inducible NOS (macrophages) and endothelial NOS *in vitro* (aorta) and *in vivo* (blood pressure). These observations show that ibuprofen arginate provides, in one preparation, a COX-2 inhibitor and NOS substrate that could act to negate the harmful cardiovascular consequences mediated by blocking renal COX-2 and increased ADMA. While remarkably simple, our findings are potentially game-changing in the nonsteroidal antiinflammatory drug arena.—Kirkby, N. S., Tesfai, A., Ahmetaj-Shala, B., Gashaw, H. H., Sampaio, W., Etelvino, G., Leão, N. M., Santos, R. A., Mitchell, J. A. Ibuprofen arginate retains eNOS substrate activity and reverses endothelial dysfunction: implications for the COX-2/ADMA axis.

Nonsteroidal antiinflammatory drugs (NSAIDs) are the most commonly taken over-the-counter medications for the treatment of pain and inflammation, with ibuprofen representing a significant share of the market. All NSAIDs work by blocking cyclooxygenase (COX) enzymes. COX has 2 isoforms: a constitutive form, COX-1, which is ubiquitously expressed, and COX-2, which is an inducible isoform rapidly expressed at the site of inflammation and in cancer. However, COX-2 is expressed constitutively without inflammation in discrete regions of the body (1, 2). These areas include the brain, thymus, gut, and kidney (1, 2). Inhibition of inducible COX-2 activity explains most of the therapeutic benefit of NSAIDs (antiinflammatory, analgesic, antipyretic); however, inhibition of constitutively expressed COX-2 by NSAIDs, including ibuprofen, causes adverse effects that limit their use (3). Upper gastrointestinal adverse effects associated with NSAID usage are due to the combined inhibition of COX-1 and -2 (4) but are relatively well managed by the use of selective COX-2 NSAIDs and coadministration of proton pump inhibitor drugs. However, NSAID-associated cardiovascular effects remain a major clinical concern. Initially, authorities such as the American Heart Association considered cardiovascular toxicity to be an issue mainly for COX-2 selective NSAIDs (5). However, because all NSAIDs work by blocking COX-2 (6), we and others suggested that cardiovascular toxicity is a class effect (7), an idea that is now universally accepted (8). The risk of cardiovascular adverse effects associated with blocking COX-2 has resulted in a series of regulatory events, including: *1*) the withdrawal of the blockbuster drug rofecoxib (Vioxx) in 2004, *2*) the introduction of black box warnings on some NSAIDs from 2005 and on all drugs in this class since 2015, *3*) the withdrawal of celecoxib (Onsenal) for the prevention of cancer in 2011, and *4*) the reclassification of the over-the-counter medication diclofenac as prescription only in 2015 (UK). Now the fear of cardiovascular events caused by NSAIDs has resulted in the cautious prescribing of COX-2 selective drugs (9) in favor of older-style medications, which are more toxic to the gut, and a failure to realize the full clinical potential of NSAIDs in the prevention of cancer (10).

While the mechanisms that mediate cardiovascular side effects of blocking COX-2 are incompletely understood, they are thought to involve blocking the release of cardioprotective lipid mediators (prostanoids) including prostacyclin from endothelial cells (11) or in the kidney (12, 13). Our recent work has shown that constitutively expressed COX-2 in the kidney provides a powerful regulation of the methylarginine system (12). Methylarginines, such as l-*N*^G^-monomethyl arginine (l-NMMA) and asymmetric dimethylarginine (ADMA), are naturally occurring molecules formed when arginine residues in proteins are methylated and subsequently broken down (14, 15). l-NMMA and ADMA act as competitive inhibitors with the natural substrate l-arginine at the active site of the enzyme NOS (15). In this way, l-NMMA and ADMA can compromise systemic cardiovascular health by blocking the NO release in blood vessels that contributes to vasodilation, platelet inhibition, and protection against atherosclerosis. Our work strongly indicates ADMA acts as a biomarker and mechanistic bridge between inhibition of COX-2 by NSAIDs and vascular dysfunction. We found increased plasma ADMA levels in COX-2-deficient mice and in wild-type mice or healthy human volunteers treated with COX-2 inhibitors (12). ADMA is also increased in the plasma of patients with arthritis (16), the majority of whom will be taking NSAIDs (16, 17). This idea has credibility within the cardiovascular field because ADMA is an established cardiovascular biomarker in both preclinical and clinical studies and, importantly, is associated with some indicators of cardiovascular disease (18) and death (19) in large cohort studies such as the Framingham Heart Study. Importantly, if our hypothesis is correct, the cardiovascular toxicity in susceptible NSAID users could be prevented by administration of l-arginine, which would counteract the effects of ADMA through competition for the active site of NOS.

Ibuprofen is one of the most commonly used nonprescription NSAIDs, and this is set to increase with the reclassification of over-the-counter diclofenac (United Kingdom) and with the virtual arrest (due to concerns over adverse effects) of new drug development in this class. Ibuprofen is relatively slowly absorbed in the gut, but several formulations have been developed to accelerate absorption, including combining ibuprofen as a salt with basic amino acids such as lysine (20) and arginine (21). Pharmaceutical ibuprofen arginate marketed by Zambon Pharma (Lonigo, Italy) under the trade names Spididol, Spedifen, Espididol, and Faspic takes the form of a preparation with ≈1:1 molar ratio of ibuprofen and arginine. As with all salts, once in solution, ibuprofen and arginine will dissociate, and so ibuprofen arginate should deliver arginine to function in an identical manner to other sources of arginine. At the maximum recommended doses of ibuprofen of up to 2.4 g/d, this would equate to administration of ∼2 g of arginine, an amount that has been shown to produce beneficial cardiovascular effects in humans (22). As such, in addition to its improved pharmacokinetics *vs.* free ibuprofen, ibuprofen arginate has the potential to provide an effective COX-2 inhibitor and substrate for NO in one clinically approved pharmaceutical preparation. While ibuprofen arginate has, in a single previous study, been shown to release NO (23), the idea that it can substitute for l-arginine and/or reverse the effects of substrate inhibitors in a setting of NSAID cardiovascular toxicity has simply not been previously considered.

To explore this idea, we systematically studied the 2 putative pharmacological possibilities of ibuprofen arginate—inhibition of COX-2 and rescue of the NOS pathway—in conditions where extracellular arginine is low and where intracellular arginine is limited by the presence of substrate inhibitors including ADMA, l-NMMA, and l-nitro-arginine methyl ester (l-NAME).

## MATERIALS AND METHODS

### *In vitro* studies

RAW264.7 cells and A549 cells were purchased from American Type Culture Collection (Manassas, VA, USA) and cultured in either arginine-free DMEM (Thermo Fisher Scientific Life Sciences, Waltham, MA, USA) or standard DMEM (containing 400 µM l-arginine; Sigma-Aldrich, St. Louis, MO, USA), both supplemented with fetal calf serum (10%; LabTech, Uckfield, United Kingdom). Confluent cultures were treated with LPS (RAW264.7 cells; 1 μg/ml; Sigma-Aldrich) or IL-1β (A549 cells; 10 ng/ml; R&D Systems, Minneapolis, MN, USA) for 24 h to induce iNOS and COX-2, in some cases in the presence of the NOS inhibitors, l-NAME (300 μM; Sigma-Aldrich) or ADMA (1 mM; Sigma-Aldrich). Increasing concentrations of ibuprofen arginate (Zambon Pharma), ibuprofen sodium (Sigma-Aldrich), l-arginine free base (Sigma-Aldrich), or the combination of ibuprofen sodium and l-arginine were also added such that the molar concentration of l-arginine present in each preparation was the same, or for ibuprofen sodium, the molar concentration of ibuprofen. After 24 h, media were collected for measurement of nitrite accumulation using the Griess reaction (Sigma-Aldrich) or PGE_2_ using immunoassay (Cisbio, Codolet, France). Cell viability was assessed using the alamarBlue metabolic activity assay (Thermo Fisher Scientific).

### Animals

Experiments were performed on male 8-wk-old C57BL/6 mice and Wistar Kyoto rats, both bred at the Biologic Science Institute (CEBIO, Federal University of Minas Gerais, Belo Horizonte, Brazil). Animals were housed with a 12 h light/dark cycle with free access to water and standard rodent chow. All animal experiments were conducted in line with EU directive 2010/63/EU and subject to ethical review by the Imperial College Ethical Review Board (PPL 70/8422) and/or the Federal University of Minas Gerais Animal Care Committee.

### Isolated vessel studies

Mice or rats were humanely killed by cervical dislocation and the thoracic aorta carefully removed and cleaned of periadventitial fat. Mouse and rat aortas were loaded into organs baths containing Krebs buffer (composition: 120 mM NaCl; 4.7 mM KCl; 1.2 mM MgSO_4_; 1.2 mM KH_2_PO_4_; 25 mM NaHCO_3_; 0.03 mM EDTA; 5.5 mM d-glucose; Sigma-Aldrich) at 37°C. Diclofenac (10 μM; Novartis, Basel, Switzerland) was added to the Krebs buffer to block vascular prostanoid formation that could confound interpretation of the effect of arginine preparations. Changes in force under a resting tension of ∼10 mN were recorded *via* an isometric force transducer (mouse aorta; DMT, Aarhus, Denmark; rat aorta, World Precision Instruments, Sarasota, FL, USA) connected to a digital signal acquisition system (ADInstruments, Dunedin, New Zealand).

Aortas were precontracted with a submaximal concentration of phenylephrine (mouse 100 nM; rat 60 nM; Sigma-Aldrich). For mouse vessels, l-NAME (200 μM) was then added to produce contraction driven by reduced NO caused by arginine insufficiency. Once the resulting l-NAME-induced contraction had stabilized, increasing concentrations of either ibuprofen arginate (0.4–2 mM) or an equimolar amount of ibuprofen sodium was added to the bath. For rat vessels, after precontraction, acetylcholine (10 μM; Sigma-Aldrich) was added to stimulate endothelial NO production. For reversal protocols in rat vessels, the acetylcholine-induced dilation was blocked by addition of l-NAME (100 μM), and once the response stabilized, increasing concentrations of either ibuprofen arginate (0.4–2 mM) or an equimolar amount of ibuprofen sodium were added to the bath. For prevention protocols in rat vessels, either ibuprofen arginate (1 mM) or ibuprofen sodium (1 mM) were added to the bath and incubated for 5 min before the addition of increasing concentrations of l-NAME (1–100 μM).

### *In vivo* blood pressure experiments

Rats were anesthetized with urethane (1.2 g/kg i.p.; Sigma-Aldrich) and tracheotomized. The left femoral artery was cannulated with polyethylene tubing and connected to a pressure transducer and signal acquisition system (Biopac Systems, Golta, CA, USA) for continuous arterial pressure recording. The left femoral vein was cannulated for administration of all subsequent drugs. Diclofenac (1 mg/kg) was administered to remove confounding effects of vascular prostanoid formation and blood pressure allowed to stabilize for 10 min. For prevention protocols, saline, l-arginine free base, ibuprofen sodium, or ibuprofen arginate were administered (each at 57.4 μmol/kg; equivalent to 10 mg/kg l-arginine, assuming full dissociation), and after 10 min, increasing concentrations of l-NAME (0.03–30 mg/kg; 3 min interval) were given. For reversal protocols, l-NAME (0.5 mg/kg) was administered and the pressor effect allowed to stabilize for 5 min before the ability of ibuprofen sodium or ibuprofen arginate (each at 114.8 μmol/kg; equivalent to 20 mg/kg l-arginine assuming full dissociation) to reverse this was determined.

### Statistical and data analysis

Data are presented as means ± sem for *n* experiments. Data were analyzed using 1- or 2-way ANOVA with Sidak’s *post hoc* test as indicated in the individual figure captions, and data were considered significant at *P* < 0.05. Analysis was performed by GraphPad Prism software (GraphPad Software, La Jolla, CA, USA).

## RESULTS AND DISCUSSION

**Figure 1.**
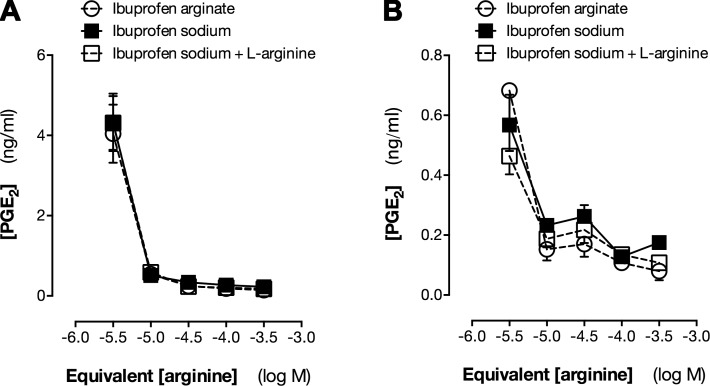
Ibuprofen arginate inhibits COX-2 in mouse (*A*) and human (*B*) cells. PGE_2_ release from mouse RAW264.7 cells stimulated with LPS (1 µg/ml) (*A*) and human A549 cells stimulated with IL-1β (10 ng/ml) (*B*) to induce COX-2. Data are presented as means ± sem for *n* = 4. *P* > 0.05 by 2-way ANOVA for all comparisons.

NSAIDs, including ibuprofen, owe their therapeutic effects to inhibition of COX-2. In order to first define the NSAID properties of ibuprofen arginate, we compared its potency for COX-2 inhibition to ibuprofen sodium. Using COX-2 induced in murine macrophages (24) (RAW264.7 cells) and human lung epithelial cells (25–28) (A549 cells) treated with inflammatory stimuli (LPS or IL-1β respectively), we observed that ibuprofen arginate had no effect on cell viability at concentrations up to 300 μM) (percentage control: RAW264.7, 119.2 ± 1,5; A549, 103.0 ± 2.1) and was equipotent to ibuprofen sodium on a molar basis for inhibiting human and mouse COX-2-dependent PGE_2_ formation (**Fig. 1**). This is consistent with the free dissociation of ibuprofen arginate to ibuprofen and arginine components in solution.

We next examined the ability of the arginine component of ibuprofen arginate to provide substrate for NOS. In plasma, arginine is present within the range of 50 to 100 μM (29) but can be >40-fold greater within vascular tissues (30). As a result of this, under normal conditions, arginine is not rate limiting for NO production (30–34). However, free arginine levels can become rate limiting under conditions where arginine is absent (35) or depleted (31), or in the presence of substrate inhibitors including ADMA (15), l-NMMA (endogenous methylarginines), and l-NAME (a synthetic inhibitory arginine analog) (34–36).

**Figure 2.**
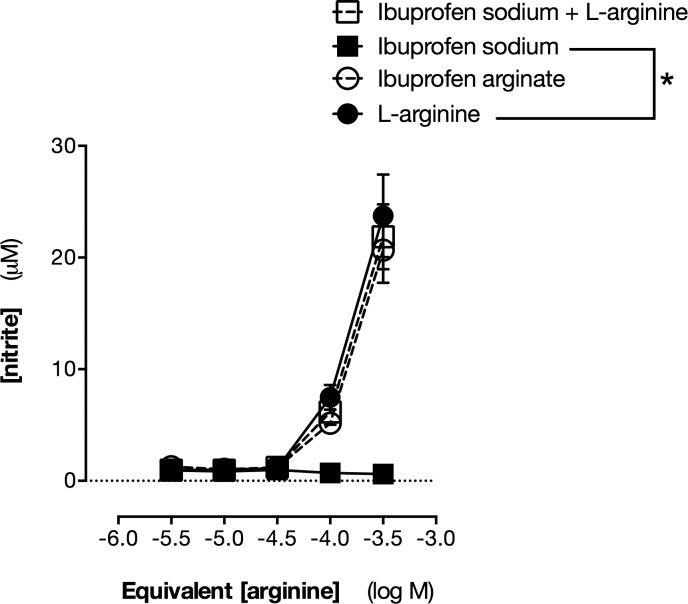
Ibuprofen arginate serves as substrate for NO production. NO production was measured as nitrite accumulation from mouse RAW264.7 cells cultured in arginine-free medium and stimulated with LPS (1 µg/ml) to induce iNOS. Data are presented as means ± sem for *n* = 4. *P < 0.05 *vs*. l-arginine by 2-way ANOVA with Sidak’s posttest.

Mouse macrophages express iNOS when stimulated with LPS in culture, and this can be quantified by measuring the formation of nitrite (35). As expected, mouse macrophages released high levels of PGE_2_ (Fig. 1) and nitrite (23.1 ± 1.6 μM) when stimulated with LPS in regular DMEM. Because DMEM (like most culture media) contains relatively high levels of arginine, ∼400 μM, iNOS is saturated with excess substrate in this system. However, when arginine was removed from the medium, nitrite was negligible (**Fig. 2**). Nitrite release could be restored by the addition of arginine in the form of arginine HCl or in the form of ibuprofen arginate (Fig. 2). Addition of ibuprofen sodium, however, had no effect on nitrite levels when given to LPS-stimulated mouse macrophages in arginine-free medium (Fig. 2). This directly demonstrates that the arginine delivered by ibuprofen arginate is able to act as substrate for NOS in the absence of other substrate.

**Figure 3.**
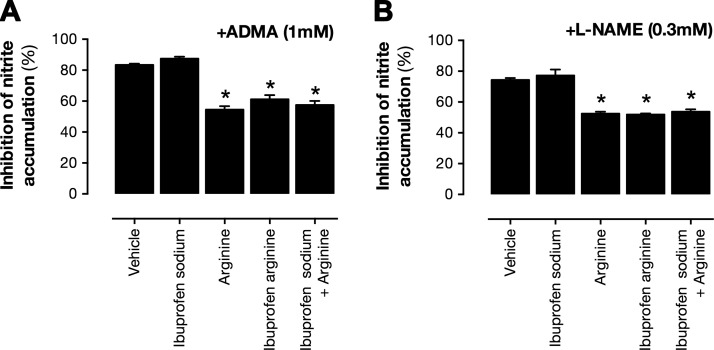
Ibuprofen arginate reverses inhibition of iNOS produced by l-NAME (*A*) and ADMA (*B*). NO production was measured as nitrite accumulation from mouse RAW264.7 cells cultured in arginine-replete medium and stimulated with LPS (1 µg/ml) to induce iNOS in presence of ADMA (*A*) or l-NAME (*B*). Data are presented as means ± sem for *n* = 4. *P < 0.05 *vs*. vehicle by 1-way ANOVA with Sidak’s posttest.

We next studied whether ibuprofen arginate could rescue NOS activity in the presence of naturally occurring (ADMA, l-NMMA) or synthetic (l-NAME) substrate inhibitors through competition of the active site of iNOS. These NOS inhibitors—ADMA, l-NMMA, and l-NAME—produced concentration-dependent reductions in nitrite levels released by LPS-stimulated mouse macrophages when cultured in normal, arginine-replete culture medium (data not shown). In each case, supplementation of the medium with arginine HCl or ibuprofen arginate, but not ibuprofen sodium, reduced the efficacy of ADMA, l-NMMA, and l-NAME (**Fig. 3**), demonstrating that in an iNOS system, ibuprofen arginate can rescue NO dysfunction produced by endogenous NOS inhibitors.

**Figure 4.**
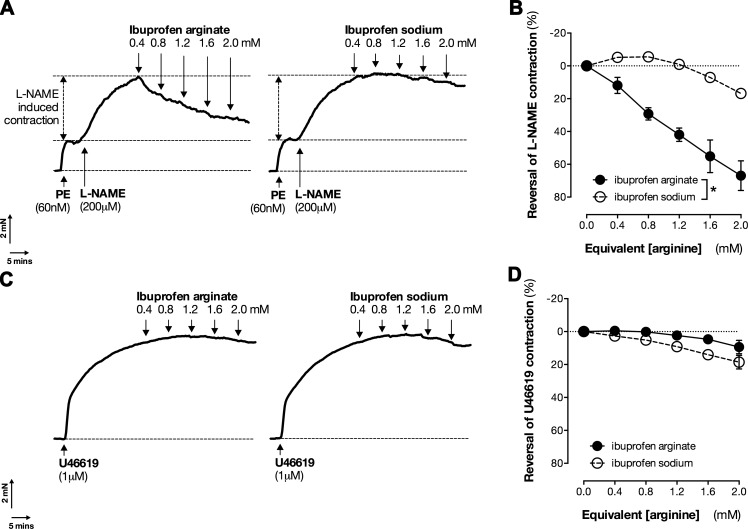
Ibuprofen arginate reverses contraction induced by eNOS inhibition (*A, B)* but not thromboxane (*C, D*) in mouse aorta. Contraction of mouse aortic rings induced by l-NAME after phenylephrine (PE) precontraction (*A, B*) or induced by U46619 (*C, D*). *A*, *C*) Representative traces. *B*, *D*) Quantified data. Data are presented as means ± sem for *n* = 4–8 vessels from 4 mice. *P < 0.05 by 2-way ANOVA.

In blood vessels, where NO acts as a cardioprotective mediator, NO is synthesized from arginine by the endothelial form of NOS (eNOS) (37), which is highly enriched in endothelial cells (37). Unlike iNOS, eNOS requires calcium for activation (37). To profile the ability of the arginine function of ibuprofen arginate to drive eNOS activity, we used 2 well-defined eNOS bioassay systems. First, we used mouse aorta. After stimulation with the contractile agonist phenylephrine to provide tone, vessels were treated with l-NAME, which produces a further contraction of the vessel in response to inhibition of basal NO release through eNOS. Within this system, arginine supplementation competes with l-NAME to restore basal NO release. We found that ibuprofen arginate, but not ibuprofen sodium, produced concentration-dependent reversal of the contraction caused by l-NAME (**Fig. 4**). This was a specific effect on l-NAME-induced contraction because neither ibuprofen arginate nor ibuprofen sodium reversed contraction of mouse aorta induced the thromboxane mimetic U46619 (Fig. 4).

**Figure 5.**
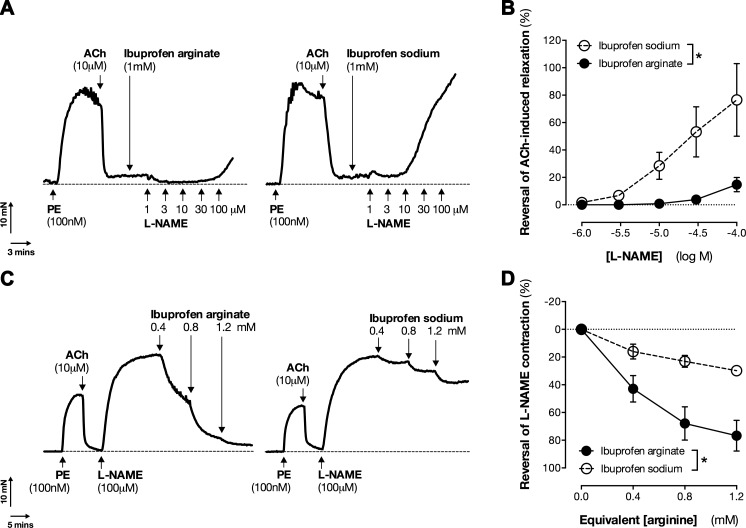
Ibuprofen arginate prevents (*A, B*) and reverses (*C, D*) l-NAME-induced endothelial dysfunction in rat aorta. Rat aortic rings were stimulated to produce NO by acetylcholine, and then ibuprofen formulations were added either before (*A, B*) or after (*C, D*) l-NAME. *A*, *C*) Representative traces. *B*, *D*) Quantified data. Data are presented as means ± sem for *n* = 4 rats. *P < 0.05 by 2-way ANOVA.

Second, we used a classic pharmacologic bioassay for demonstration of eNOS activity (38). In rat aorta contracted with phenylephrine to provide a basis for vasodilation, the endothelium was stimulated with acetylcholine to activate eNOS, which produced an almost complete antagonism of the contraction caused by phenylephrine. In this model, because arginine is in excess within the tissue, addition of exogenous arginine has minimal effect on eNOS responses. However, intracellular levels of arginine can be made rate limiting by the addition of arginine substrate inhibitors such as l-NAME. In this setting, addition of l-NAME can produce a complete reversal of the eNOS-driven relaxation as a result of its competition with arginine for the active site of eNOS (39). This reflects a situation within the endothelium comparable to an arginine-free environment in which eNOS activity can be revealed by the subsequent addition of arginine (39). Under these conditions, preincubation of vessels with ibuprofen arginate but not ibuprofen sodium reduced the ability of l-NAME to reverse acetylcholine-induced vasodilation of phenylephrine precontracted rat aortic rings (**Fig. 5*A, B***). Correspondingly, when added after l-NAME, addition of ibuprofen arginate but not ibuprofen sodium was able to reverse an established contraction of rat aortic rings driven by arginine insufficiency (Fig. 5*C, D*). These data firmly demonstrate that ibuprofen arginate can restore eNOS, as well as iNOS activity in situations where substrate availability is limited *in vitro*.

**Figure 6.**
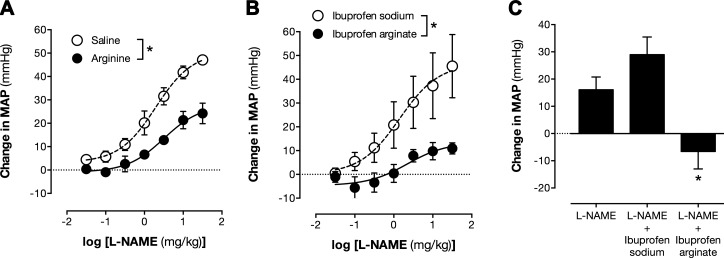
Ibuprofen arginate prevents and reverses acute l-NAME-induced pressor responses in rat *in vivo*. l-NAME-induced arterial blood pressure increases in anesthetized rats where ibuprofen/arginine formulations are administered either before (*A, B*) or after (*C*) l-NAME. Data are presented as means ± sem for *n* = 3–4 rats. *P < 0.05 by 2-way ANOVA (*A, B*) or 1-way ANOVA (*C*).

Last, we studied whether ibuprofen arginate exhibits similar arginine substrate activity for eNOS responses when administered *in vivo*. As in isolated vessels, arginine concentrations *in vivo* are not rate limiting for vascular eNOS activity. Therefore, to test arginine substrate activity of ibuprofen formulations *in vitro,* we produced experimental arginine insufficiency by administration of l-NAME to anesthetized rats. This produces a predictable increase in blood pressure that is driven by loss of eNOS-dependent vasodilator tone and is sensitive to arginine supplementation (40). In this model, pretreatment of rats with l-arginine free base or ibuprofen arginate, but not ibuprofen sodium, prevented l-NAME-induced pressor responses (**Fig. 6*A, B***). Similarly, when administered to rats that were first treated with l-NAME, ibuprofen arginate but not ibuprofen sodium could reverse the established blood pressure increase. Taken together, these observations demonstrate that ibuprofen arginate can support eNOS activity both *in vitro* and *in vivo.*

Thus, ibuprofen arginate in solution serves as both an effective inhibitor of COX-2 and a substrate for NOS *in vitro* and *in vivo*. In our previous work where we found COX-2 inhibition increases plasma ADMA, we also found that this resulted in reduced eNOS responses in the systemic circulation that could be rescued by the addition of l-arginine (12). We suggested that l-arginine supplementation could therefore provide an effective rescue therapy for COX-2 inhibitor cardiovascular toxicity and that a prospective clinical trial would be needed to validate our hypothesis (12). However, such a trial would be expensive, difficult to power, and would involve lengthy considerations regarding formulations. Our findings here are not surprising because ibuprofen arginate is a simple salt that in solution will dissociate into ibuprofen and arginine. Nonetheless, because ibuprofen arginate was formulated for its pharmacokinetic benefits (over ibuprofen acid), considerations of its potential to protect from NSAID-induced cardiovascular toxicity have simply never been considered before.

Thus, while remarkably simple, our findings that ibuprofen arginate has that ability to rescue eNOS dysfunction, such as might be produced by ADMA after inhibition of renal COX-2 by the NSAID component of this medication, have the potential to be globally game changing in the NSAID arena. As such, we suggest that ibuprofen arginate—an approved, clinically used, and freely available NSAID formulation—has great promise to spare the cardiovascular toxicity associated with use of drugs that inhibit COX-2. The next steps to translate this idea to the clinic would be to perform experiments in animal models of cardiovascular disease known to be sensitive to COX-2 inhibition, such as atherosclerosis (41) and thrombosis (42), and to compare the effects of ibuprofen arginate with ibuprofen sodium in healthy human subjects where arginine, ADMA, and endothelial/platelet function are measured.

## Abbreviations

ADMA: asymmetric dimethylarginine
COX: cyclooxygenase
eNOS: endothelial form of NOS
l-NAME: N^G^-nitro-l-arginine methyl ester
l-NMMA: l-*N*^G^-monomethyl arginine
NSAID: nonsteroidal antiinflammatory drug
